# Cost-effectiveness of Levodopa-Carbidopa Intestinal Gel in treating people with Advanced Parkinson’s disease

**DOI:** 10.12968/bjnn.2023.19.4.140

**Published:** 2023-08-31

**Authors:** Sonia Raj, Rekha Sarvankar, Luís Filipe, Valerio Benedetto, Nicola Mason, Jennifer Dawber, James Hill, Andrew Clegg

**Affiliations:** 1 Lancashire Teaching Hospitals NHS Foundation Trust; 2 Faculty of Health and Medicine, Lancaster University; 3 NIHR Applied Research Collaboration North West Coast (ARC NWC), Methodological Innovation, Development, Adaptation and Support (MIDAS) Theme; 4 Applied Health Research hub, University of Central Lancashire

**Keywords:** Advanced Parkinson’s disease, levodopa/carbidopa intestinal gel, costs, economic evaluation, cost-effectiveness

## Abstract

Advanced Parkinson’s disease affects patients with existing Parkinson’s disease by further deteriorating their physical and cognitive functions. In this commentary we critically assess an economic evaluation which compared the cost-effectiveness of levodopa/carbidopa intestinal gel against standard of care in treating patients with Advanced Parkinson’s disease. While the economic evaluation indicated that levodopa/carbidopa intestinal gel could be cost-effective within the UK parameters, we highlight important limitations related to its design, modelling and analysis. Future research should consider the incorporation of a separate arm dedicated to the re-infusion of apomorphine on eligible Advanced Parkinson’s disease patients, a wider set of levodopa/carbidopa intestinal gel adverse events and related costs, and a sub-group analysis on different socio-economic strata.

## Introduction

### Prevalence and impact for the individuals

Advance Parkinson’s disease is a late stage of Parkinson’s disease that is marked by limited mobility (Gilbert and Khemani 2022). As the disease progresses, people may have difficulty walking and talking (Lindh-Rengifo et al. 2021; Skodda et al. 2013). They may also have mental and behavioural changes, sleep problems, depression, memory difficulties, and fatigue (Friedman and Friedman 1993; Schrag 2004; Zakharov et al. 2001; Zuzuárregui and During 2020). Approximately 145,000 people were predicted to be living with Parkinson’s disease in the UK in 2018, with this estimate predicted to increase to approximately 256,000 by 2065 (Parkinson’s UK 2018). Of these, approximately 10% will have Advanced Parkinson’s disease (Stefani et al. 2022).

### Impact for healthcare system

The costs of Advanced Parkinson’s disease substantially increase as the proportion of a waking day spent by patients in OFF-time (i.e. when the treatment effect disappears) rises (Findley et al. 2011). Findley et al. (Findley et al. 2011) estimated that the total annual costs may rise from £25,630 when patients spend less than a quarter of their waking day in OFF-time to £62,147 when they spend more than three quarters of their waking day in OFF-time. Costs are mainly driven by the provision of professional care, which ranges from 35% to 77% of the total costs across different OFF states (Findley et al. 2011).

### Justification for economic evaluation

Given the clinical and cost impacts of Advanced Parkinson’s disease, there emerges the need for cost-effective interventions focusing on improving the conditions of patients and the burden on the NHS. In this sense, the authors of the economic evaluation which is being assessed here (Chaudhuri et al. 2022) aimed to determine the cost-effectiveness of levodopa/carbidopa intestinal gel (LCIG) in comparison to standard of care in the treatment of Advanced Parkinson’s disease patients.

### Aim of commentary

This commentary aims to critically appraise the methods and the results reported in the economic evaluation by (Chaudhuri et al. 2022) and expand upon the findings in the context of clinical practice.

## Methods

In the economic evaluation by (Chaudhuri et al. 2022), the authors assessed the cost-effectiveness of LCIG in comparison with standard of care in treating patients with Advanced Parkinson’s disease by adopting an NHS England and Personal and Social Services perspective. The treatment group consisted of patients receiving LCIG treatment, while the comparator group comprised patients who were eligible for LCIG but continued with standard of care treatment (i.e. oral medications or apomorphine retrial). The authors used a Markov model to estimate the incremental cost-effectiveness ratio, which involved determining the average costs, life years, and quality-adjusted life years (QALYs) per patient for both comparators.

The Markov model classified patients into 25 health states based on their Hoehn and Yahr (HY) stage (1 to 5, with higher stages implying more severity) and the amount of OFF-time experienced (measured in increasingly deteriorating levels from 0 to IV, assuming a 16-hour waking day), as well as a death state. Patients’ clinical trajectories were extrapolated over a period of 20 years with 6-month cycles, and a discount rate of 3.5% was applied to costs and effects.

To estimate the initial treatment effect of LCIG on patients’ HY stage and OFF-time, data from the randomised controlled trial by Fernandez et al. (Fernandez et al. 2015) was used. In particular, the cost-effectiveness model included patients with Advanced Parkinson’s disease (N=196) who experienced severe motor fluctuations (HY ≥ Stage 3 and OFF-time > 25% of a 16-hour waking day).

Utility estimates were computed to capture patients’ disutility from HY stages and OFF-time as well as caregivers’ disutility associated to patients’ HY stages. The estimates were based on the EuroQol-5-Dimension-3-Level values mapped to the UK-specific tariff (Dolan 1997) and derived from a combination of studies (AbbVie 2011; Antonini et al. 2017; Antonini et al. 2015; Fernandez et al. 2015; Olanow et al. 2014). All-cause mortality was also accounted for (Office for National Statistics 2019).

The model considered both medical and non-medical costs, including hospitalisations, emergency room visits, consultations, professional caregiving, and respite care. Health-state costs were estimated using regression analysis. Professional care accounted for a significant proportion of the total costs, with the cost of nursing home staff being the most significant factor.

To assess the impact of uncertainties surrounding the core model outputs, scenario analyses and one-way sensitivity analyses were conducted. A probabilistic sensitivity analysis was also conducted to assess the uncertainty related to the values of the model parameters and to test the model’s robustness.

## Results

The base-case analysis showed that the LCIG cohort had (on average) 0.36 more life years and 1.39 more QALYs compared with the cohort who received standard of care (10.64 vs 10.28 life years and 2.82 vs 1.43 QALYs, respectively).

On the other hand, the average costs were higher for the LCIG cohort (£586,832) than for the standard of care cohort (£554,022).

As a result, the incremental cost-effectiveness ratio for LCIG over standard of care was £90,349 per life years gained and £23,649 per QALY gained.

The cost-effectiveness acceptability curve indicated the probability of LCIG being cost-effective at different willingness-to-pay thresholds per QALY gained. This probability was below 20% with a willingness to pay equal to £0, then increased to 40% with a willingness to pay of £20,000 and to 55% with a willingness to pay of £30,000.

Deterministic one-way sensitivity analyses showed that variations in LCIG long-term efficacy after the first year (risk ratio equal to 0 or 1), health state costs (± 20%), long-term LCIG discontinuation rate after the first year (5% or 15% annually) were associated with the bigger changes in the incremental net monetary benefit for a willingness to pay of £30,000 per QALY gained. Scenario analyses based on an alternative population (HY = Stage 3+/OFF-time = Level III+) and on the assumption that 20% of the standard of care cohort would be administered apomorphine highlighted a positive change in incremental net monetary benefit in favour of LCIG. In addition, the base-case analysis results were robust to changes in long-term LCIG efficacy after 5 years.

The probabilistic sensitivity analysis plotted different combinations of possible cost-effectiveness pairs on a cost-effectiveness plane. The majority of pairs lay on the north-east quadrant, thus indicating a greater likelihood of detecting higher costs and QALYs for LCIG (compared with standard of care). The remaining pairs lay on the south-east quadrant, which would mean lower costs and higher QALYs for LCIG. The authors indicated that this variability was mainly due to the range of values that health-state costs can take, which in turn can change the quadrant where the cost-effectiveness points lie (and consequent interpretation).

In conclusion, the authors stated that the base-case analysis showed an incremental cost-effectiveness ratio of £23,649 per QALY gained which would fall within the cost per QALY threshold typically used in the UK (between £20,000-30,000). This was driven by the higher health-related quality of life and treatment costs associated with the LCIG cohort compared with the standard of care cohort.

## Commentary

### Critical appraisal

We assessed the quality of the economic evaluation by Chaudhuri et al. (Chaudhuri et al. 2022) by using a hybrid tool which combined different questions from existing tools (Critical Appraisal Skills Programme 2018; Drummond et al. 2015; Philips et al. 2004), and we identified issues with 6 areas (out of 16 areas we examined – see Table 1).

First, the rationale behind the option of retrying the infusion of apomorphine in standard of care patients who are initially unsuitable was unclear. It also seems unclear how many patients would be eligible to this option. In this sense, the addition of a separate arm specifically dedicated to patients who are eligible to re-infusion of apomorphine could have been instrumental in estimating the specific effects that this treatment option may have on Advanced Parkinson’s disease patients.

Second, the authors used effectiveness data which was arbitrarily sourced from a set of studies, and not through a purposely developed systematic review of the relevant literature.

Third, the economic evaluation did not seem to consider relevant adverse events and complications related to LCIG (such as infections, blocked tube, or injury on abdominal area) which could trigger additional costs in terms of use of antibiotics and longer hospital stay.

Fourth, the authors applied a mortality probability adjustment for different HY stages in their model, but did not apply a similar adjustment for different OFF-time levels nor a joint mortality probability adjustment for different combinations of HY stages and OFF-time levels.

Fifth, the authors did not explore how the results varied according to different socio-economic and demographic characteristics of Advanced Parkinson’s disease patients which could have revealed differential cost-effectiveness levels of LCIG on different sub-groups.

Sixth, given the above limitations which affected the design, modelling, and analysis of the economic evaluation, the authors seemed to overstate the potential cost-effectiveness of the LCIG in their conclusions.

Moreover, the authors declared conflicts of interest with respect to their connections to a pharmaceutical company involved in the production of LCIG.

### Implications for practice

LCIG is a surgical procedure that carries a high risk of complications, including the potential for multiple infections (Udd et al. 2017). Additionally, it is expensive, complex to administer and requires intensive monitoring (De Fabregues et al. 2017). As such, it should be considered a last resort option and only utilised when all other options have been exhausted (Lowin et al. 2011). Considering this study’s lack of clarity regarding the use of apomorphine as the standard of care, healthcare practitioners should first attempt different dosages of apomorphine if the patient is able to tolerate it (NHS England 2015).

If LCIG is ultimately chosen, the use of opicapone may reduce the likelihood of requiring a second LCIG cassette (Leta et al. 2020). While LCIG is more complex, it can serve as a helpful alternative in some cases, as it eliminates the need for regular needle changes and the pain associated with administering apomorphine (Carbone et al. 2019).

### Recommendations for future research

To improve comparability in future research, it would be beneficial to demonstrate the effects of re-infusion of apomorphine on Advanced Parkinson’s disease patients as a separate arm. In the cost-effectiveness model, joint mortality probability adjustments should be utilised for different combinations of HY stages and OFF-time levels. To enhance the analysis and provide a deeper understanding of patients’ conditions, the use of Unified Parkinson’s Disease Rating Scale score (Fahn et al. 1987) should be considered as a potential complement to HY and OFF-time interactions.

When evaluating the costs associated with LCIG, adverse events such as infections should be included in the estimations of costs to avoid underestimating the costs and overestimating the cost-effectiveness of LCIG. Also, future economic evaluations based solely on secondary data should rely on systematic reviews to ensure that the most relevant studies inform the decision modelling and corresponding parameters. Lastly, it would be insightful to explore potential barriers to the widespread adoption of LCIG in healthcare services, as well as the cost-effectiveness of LCIG changes across different socio-economic sub-groups.

### CPD reflective questions

1. Do you agree with the conclusions of the economic evaluation in terms of the cost-effectiveness of levodopa/carbidopa intestinal gel (LCIG) compared with standard of care? Why?
2. Do you think that the economic evaluation included all the relevant costs or outcomes? Why?
3. Which barriers and facilitators can you identify in the administration of LCIG in patients with Advanced Parkinson’s disease?

## Figures and Tables

**Table 1:** Critical appraisal tool

#	Question	Answer
	**A. Rationale**	
**A1**	**Is there a clear statement of the decision problem?**	☐ Yes ☐ No ☒ Unclear ☐ Not applicable
	**B. Effectiveness**	
**B1**	**Was the effectiveness of the intervention established on a systematic review?**	☐ Yes ☒ No ☐ Unclear ☐ Not applicable
	**C. Comparators**	
**C1**	**Was a comprehensive description of the competing alternatives given? (i.e. can you tell who did what to whom, where, and how often)**	☒ Yes ☐ No ☐ Unclear ☐ Not applicable
	**D. Model perspective and structure**	
**D1**	**Is the perspective of the model clearly stated?**	☒ Yes ☐ No ☐ Unclear ☐ Not applicable
**D2**	**Are the model structure and its assumptions appropriate and do they fit with the clinical theory of the disease process?**	☒ Yes ☐ No ☐ Unclear ☐ Not applicable
	**E. Costs**	
**E1**	**Were all important and relevant costs for each alternative identified?**	☐ Yes ☒ No ☐ Unclear ☐ Not applicable
**E2**	**Were costs measured and valued appropriately?**	☒ Yes ☐ No ☐ Unclear ☐ Not applicable
	**F. Outcomes**	
**F1**	**Were all important and relevant outcomes for each alternative identified?**	☐ Yes ☐ No ☒ Unclear ☐ Not applicable
**F2**	**Were outcomes measured and valued appropriately?**	☒ Yes ☐ No ☐ Unclear ☐ Not applicable
	**G. Analysis**	
**G1**	**Was the analysis designed appropriately?**	☒ Yes ☐ No ☐ Unclear ☐ Not applicable
**G2**	**Were the methods and assumptions used to extrapolate short-term results to final outcomes been documented and justified?**	☒ Yes ☐ No ☐ Unclear ☐ Not applicable
**G3**	**Was uncertainty in the estimates of costs and outcomes adequately characterised?**	☒ Yes ☐ No ☐ Unclear ☐ Not applicable
	**H. Presentation and discussion of findings**	
**H1**	**Were the results interpreted appropriately?**	☐ Yes ☒ No ☐ Unclear ☐ Not applicable
**H2**	**Did the study discuss the generalisability of the results to other settings and patient/client groups?**	☒ Yes ☐ No ☐ Unclear ☐ Not applicable
**H3**	**Did the study allude to, or take account of, other important factors in the choice or decision under consideration (e.g. distribution of costs and consequences, relevant ethical issues, or issues of implementation)?**	☐ Yes ☒ No ☐ Unclear ☐ Not applicable
	**I. Transferability to UK NHS**	
**I1**	**Are the health care system, setting, comparator and patient group comparable to the UK and to the NHS?**	☒ Yes ☐ No ☐ Unclear ☐ Not applicable

## Key Points

1. In treating patients with Advanced Parkinson’s disease, the economic evaluation by Chaudhuri et al. (2022) showed that the use of levodopa/carbidopa intestinal gel (LCIG) could be cost-effective within the UK parameters when compared with standard of care.
2. Important limitations emerge with respect to the economic evaluation’s design (e.g. missed inclusion of an arm dedicated to the re-infusion of apomorphine on eligible patients), modelling (e.g. exclusion of LCIG adverse events and related costs) and analysis (e.g. lack of sub-group analysis on different socio-economic strata).
3. LCIG may show promise in terms of cost-effectiveness compared with standard of care but remains an expensive procedure which carries the risk of clinical complications such as multiple infections.
4. Future research should look at corroborating or challenging the results of the economic evaluations by Chaudhuri et al. (2022) and expand on the related findings by considering potentially critical clinical (e.g. adverse events) and financial implications of using LCIG in patients with Advanced Parkinson’s disease.
